# No effect of occupational noise exposure on auditory brainstem response and speech perception in noise

**DOI:** 10.3389/fnins.2022.915211

**Published:** 2022-07-22

**Authors:** Alexis Pinsonnault-Skvarenina, Karina Moïn-Darbari, Wulan Zhao, Meibian Zhang, Wei Qiu, Adrian Fuente

**Affiliations:** ^1^École d’Orthophonie et d’Audiologie, Faculté de Médecine, Université de Montréal, Montréal, QC, Canada; ^2^Centre de Recherche Interdisciplinaire en Réadaptation du Montréal Métropolitain – CIUSSS du Centre-Sud-de-l’Île-de-Montréal, Montréal, QC, Canada; ^3^Centre for Interdisciplinary Research in Music Media and Technology, McGill University, Montréal, QC, Canada; ^4^Centre de Recherche de l’Institut Universitaire de Gériatrie de Montréal – CIUSSS du Centre-Sud-de-l’ÎIe-de-Montréal, Montréal, QC, Canada; ^5^Zhejiang Chinese Medical University, Hangzhou, China; ^6^National Institute of Occupational Health and Poison Control, Beijing, China; ^7^Auditory Research Laboratory, State University of New York at Plattsburgh, Plattsburgh, NY, United States

**Keywords:** cochlear synaptopathy (CS), hidden hearing loss (HHL), occupational noise exposure, auditory brainstem response (ABR), speech perception in noise (SPiN)

## Abstract

The primary aim of this study was to investigate whether auditory brainstem response (ABR) and speech perception in noise (SPiN) were associated with occupational noise exposure in normal hearing young factory workers. Forty young adults occupationally exposed to noise and 40 non-exposed young adults (control group) from Zhejiang province in China were selected. All participants presented with normal hearing thresholds and distortion product otoacoustic emissions. Participants were evaluated with the Mandarin Bamford-Kowal-Bench (BKB) test and ABR. The latter was obtained for click stimulus at 50, 60, 70, 80, and 90 dBnHL. Peak-to-trough amplitudes and latencies for waves I and V were obtained. The ABR wave I amplitude, the wave I/V amplitude ratio, the slope of the wave I amplitude growth as a function of stimulus intensity (AMP-I_Slope_), and the wave V latency shift with ipsilateral noise (LAT-V_Slope_) were used as ABR outcomes. Finally, equivalent continuous average sound pressure level normalized to 8 h (L_Aeq.8h_) and cumulative noise exposure (CNE) were obtained for noise-exposed participants. No significant differences between groups were found for any ABR outcomes. Noise-exposed participants exhibited worse BKB scores than control group participants. A multivariate regression model showed that 23.3% of the variance in BKB scores was explained by group category (exposed vs. non-exposed) and hearing thresholds. However, since none of the ABR outcomes exploring cochlear synaptopathy were associated with noise exposure, we cannot conclude that cochlear synaptopathy was the contributing factor for the differences between groups for BKB scores. Factors that go beyond sensory processing may explain such results, especially given socio-economic differences between the noise-exposed and control groups. We conclude that in this sample of participants, occupational noise exposure was not associated with signs of cochlear synaptopathy as measured by ABR and BKB.

## Introduction

A number of studies have reported that a moderate-to-high noise exposure can induce auditory damage in experimental animals (Kujawa and Liberman, 2009; Lin et al., 2011; Furman et al., 2013; Fernandez et al., 2015; Gannouni et al., 2015; Jensen et al., 2015). While most of these studies found auditory damage after short exposures (i.e., 97–106 dB SPL for 2 h), lower exposure levels for longer duration (i.e., 70 and 85 dB SPL, 6 h/day, 3 months) have also been shown to be harmful (Gannouni et al., 2015). This auditory damage is characterized by an injury to inner hair cell (IHC) synapses (Kujawa and Liberman, 2009), with a subsequent preferential loss of low spontaneous rate (SR) auditory fibers (Furman et al., 2013; Fernandez et al., 2015). This phenomenon has been referred to as cochlear synaptopathy and has also been associated with normal aging in animals without noise exposure (Sergeyenko et al., 2013; Gleich et al., 2016). Because low-SR auditory fibers are not involved in the coding of the amplitude of low-level sounds (Ruggero, 1992; Bourien et al., 2014), an injury to such fibers does not affect hearing thresholds. In the animal model, this can be observed by a reduction in auditory brainstem response (ABR) wave I amplitude at suprathreshold levels (Kujawa and Liberman, 2009; Sergeyenko et al., 2013). Therefore, cochlear synaptopathy may be observed despite normal hearing thresholds and the integrity of outer hair cells (OHC), as measured by otoacoustic emissions (Kujawa and Liberman, 2009).

Studies investigating cochlear synaptopathy in humans *in vivo* have used behavioral and electrophysiological measures to detect auditory deficits induced by noise exposure (for a review, see Barbee et al., 2018; Leroux and Pinsonnault-Skvarenina, 2018). The ABR (at suprathreshold levels) and speech perception in noise (SPiN) tests are the most used procedures for such purposes. Regarding ABR measures, previous studies have typically investigated the amplitude of wave I. However, the wave I/V amplitude ratio (e.g., Schaette and McAlpine, 2011), the summating potential (SP)/action potential (AP) ratio from the electrocochleography (e.g., Liberman et al., 2016), the wave I amplitude growth as a function of stimulus intensity (e.g., Kujawa and Liberman, 2009), the wave V latency (e.g., Skoe and Tufts, 2018), and the shift in the latency of wave V as a function of an ipsilateral white noise masker (e.g., Mehraei et al., 2016) have been proposed for their ability to serve as biomarkers of cochlear synaptopathy. Results from different studies using these procedures with normal hearing young individuals are controversial. This is because some studies have found an association between ABR measures and/or SPiN test results and noise exposure (e.g., Liberman et al., 2016; Bramhall et al., 2017; Grant et al., 2020; Kikidis et al., 2020; Wang et al., 2021), while others have not (e.g., Fulbright et al., 2017; Grinn et al., 2017; Grose et al., 2017; Prendergast et al., 2017a,b; Yeend et al., 2017; Washnik et al., 2020). Some authors have suggested that ABR and SPiN measures may not be sensitive enough to detect this condition or that cochlear synaptopathy may not manifest in young people with normal hearing thresholds (Guest et al., 2018; Bramhall, 2021). Additionally, some authors have suggested that typical recreational noise exposure may not be sufficient to cause cochlear synaptopathy in young normal hearing individuals (Prendergast et al., 2017a; Guest et al., 2018).

Most of the previous studies have investigated samples of young adults recreationally exposed to noise. Little is known about occupational populations exposed to noise. If noise-induced cochlear synaptopathy occurs in humans, then it is likely that workers exposed to noise may develop such a condition prior to permanent threshold shifts. Indeed, it has been documented that normal hearing workers exposed to noise complain of challenges understanding speech in difficult listening situations, despite presenting with normal hearing thresholds (Soalheiro et al., 2012). Difficulties understanding speech in challenging acoustical conditions in the presence of normal hearing thresholds have been proposed as a perceptual consequence of cochlear synaptopathy (e.g., Liberman et al., 2016; Mepani et al., 2020). Thus, we hypothesize that workers exposed to noise may develop cochlear synaptopathy, and that such a condition can be detected using ABR and SPiN tasks. Identifying cochlear synaptopathy in workers exposed to noise may be key for prevention programs in this population. Accordingly, the aim of this study was to determine whether ABR results and scores for a SPiN test were associated with occupational noise exposure in young workers with normal hearing thresholds and presence of otoacoustic emissions.

## Materials and methods

The Ethics Committee of the Faculty of Medicine of the University of Montreal, the Committee on the Protection of Human Subjects of SUNY Plattsburgh, and the Ethics Committee of Zhejiang Provincial Center for Disease Control and Prevention approved the study protocol. All participants signed a consent form prior to being included in the study.

### Participants

Two groups of participants from Zhejiang province in China were selected. Forty male workers exposed to occupational noise (noise-exposed group) at or above 80 dBA (based on the equivalent continuous average sound pressure level normalized to 8 h, L_Aeq.8h_), along with 40 male participants without occupational exposure to noise (control group), were recruited. Participants from both groups were required to be aged between 18 and 40 years and have no family history of hearing loss, history of ear surgery, use of ototoxic drugs, or neurological disorders. They all presented with normal tympanometry (middle-ear pressure and compliance readings), and hearing thresholds (in at least one ear) equal to or better than 20 dB HL across frequencies (0.5–8 kHz). They also exhibited the presence of distortion product otoacoustic emissions (DPOAEs) (at least +3 dB SNR) for the frequency range of 2–10 kHz. Additionally, extended high frequency thresholds (9–14 kHz) were measured in both groups, but they were not used as an exclusion criterion. All participants were native Mandarin speakers.

### Procedures

A research team member administered a questionnaire to the participants in both groups in order to collect the following information: general demographic information (e.g., age); occupational history (e.g., factories, worksite, job description, length of employment, duration of daily noise exposure, and history of using hearing protection); and overall health status (e.g., history of ear disease or ototoxic drug exposure). Workers exposed to noise for a minimum of 2 years in the same workplace were selected from four different types of industries (furniture manufacturing, *n* = 6, 15%; industrial equipment manufacturing, *n* = 12, 30%; electric and appliances’ industry, *n* = 16, 40%; textile industry, *n* = 6, 15%) located in Zhejiang province, China. Participants without occupational noise exposure (control group) were university students from the Zhejiang Chinese Medical University. Participants with a history of ear disease or other related health conditions associated with auditory disorders were not included in the sample. Additionally, participants in both groups were asked whether they had experienced significant recreational noise exposure. This means exposure to firearms, playing a musical instrument or in a band, frequent attendance to concerts or sporting events, noisy bars and and/or nightclubs, along with excessive use of listening devices at elevated volumes. Participants’ responses to this question were used to make sure that they did not report significant exposure to recreational noise.

Selected participants were scheduled for an assessment session at Zhejiang Chinese Medical University (Hangzhou, China). Initially, bilateral otoscopy was carried out with the aim of excluding participants with abnormalities in the external ear canal and tympanic membrane. Hearing testing was conducted in a double-walled, soundproofed, and electrically shielded room. The better ear (based on the results of pure-tone audiometry and DPOAEs tests) was selected for the statistical analyses.

#### Use of hearing protection devices

The frequency of use of hearing protection devices (HPDs) in the workplace, usually slow-recovery formable earplugs, was assessed through field observations by the industrial hygienist and in the questionnaire. For those participants who had never used HPDs, the members of the research team recommended the use of appropriate HPDs after data collection. During this study, workers in the investigated factories received training on how to properly use HPDs.

#### Noise exposure assessment in participants occupationally exposed to noise

Shift-long noise recordings were obtained for each noise-exposed participant using an ASV5910-R digital recorder (Hangzhou Aihua Instruments Co., Hangzhou, China). The ASV5910-R digital recorder is a specialized sound recording device that can be used for precision measurements and analysis of personal noise exposure since it allows to record the waveform. The instrument uses a ^1^/_4_-inch pre-polarized condenser microphone characterized by good stability, a high upper measurement limit, and wide frequency response (20 Hz – 20 kHz). The sensitivity level of the microphone is 2.24 mV/Pa, and the measurement range is 40–141 dBA. The device was worn on the worker’s shoulder during the entire work shift. The recorder was calibrated before and after each sampling period with the use of a sound level calibrator (Hangzhou Aihua Instruments, AWA6221B), according to the instructions provided by the manufacturer. Before recording, a research team member confirmed with the manager of the workplace that this was the noise the workers were typically exposed to on an average working day. One full-shift recording of each participant’s noise exposure was captured by the ASV5910-R at 32-bit resolution with a 48-kHz sampling rate and saved in a raw audio format (WAV file). The noise record was saved on a 32 GB micro-SD card and transferred to a portable hard disk for subsequent analysis. The equivalent continuous average sound pressure level (LEQ) normalized to 8 h (L_Aeq.8h_) was obtained for each worker. Each one presented with a L_Aeq.8h_ equal to or higher than 80 dBA. In addition, a composite noise exposure index, the CNE, in dBA.year, was calculated to quantify the noise exposure for each participant. The CNE is defined as:


$$\text{CNE}={\text{L}}_{\mathrm{Aeq}\,.8\,h}+10\,\text{log}\,\text{T}$$

where L_Aeq.8h_ is the equivalent continuous A-weighted noise exposure level normalized to an 8-h working day, in decibels, occurring over the time interval T in years.

As can be seen in the calculation, when a noisy activity is performed for many years, the numeric contribution to the total CNE diminishes with each additional year. Therefore, the CNE considers that early exposure has contributed more to the total exposure energy because the accumulation of noise exposure over the years is logarithmic. It has been reported that noise-induced hearing loss develops most rapidly in the first 10 years and then slows with additional exposure to noise (Dobie, 2001; Zhang et al., 2020). The CNE was previously used to evaluate the risk of hearing loss in workers exposed to occupational noise (e.g., Zhao et al., 2010; Xie et al., 2016).

In addition, corrected L_Aeq.8h_ (L_Aeq.8h_-HPD) and CNE (CNE-HPD) were calculated by incorporating estimates of HPD use into individual noise exposure calculations. First, the attenuation of each participant’s HPD was derated based on the NIOSH recommendations to compensate for known differences between laboratory-derived attenuation values and the attenuation obtained in the real world (National Institute for Occupational Safety and Health [NIOSH], 1998). To do so, the noise reduction rating (NRR) was reduced by 50% since all participants used slow-recovery formable earplugs. For example, if a participant used an HPD with an attenuation of 29 dB, the derated NRR value was 14.5 dB. This value was then subtracted from the L_Aeq.8h_ of each participant. For example, if a participant presented with a L_Aeq.8h_ of 90 dBA, the 14.5 dB NRR was subtracted, and a new L_Aeq_ of 75.5 dBA was obtained. Then, the L_Aeq.8h_-HPD value was obtained for each participant, based on the frequency of HPD use. For example, if a participant reported using HPDs ∼25% of the time, the total unprotected exposure (75% of the total time at a L_Aeq_ without HPD; 6 h at 90 dBA in this example) and the total protected exposure (25% duration at a protected level; 2 h at 75.5 dBA in this example) were combined. Finally, a corrected CNE value (CNE-HPD) was calculated for each participant based on the L_Aeq.8h_-HPD. The L_Aeq.8h_-HPD is defined as:


$$L_{Aeq.8h}HPD\;\;=10\;log\;[\frac{1}{8}\left(\left(T_{unprotected}\;\;10^{L_{Aeq.8h}/10}\right)+\left(T_{protected}\;\;10^{(L_{Aeq.8h}-NRR\;\;50\%)/10}\right)\right)]$$

where L_Aeq.8h_-HPD is the equivalent continuous A-weighted noise exposure level normalized to an 8-h working day and corrected for HPD attenuation, in decibels, occurring over the time interval T_unprotected_ and T_protected_ in hours.

#### Tympanometry and pure-tone audiometry

An Interacoustics Titan device (Middelfart, Denmark) was used for tympanometry. The tympanometer probe was inserted into the external auditory canal. A 1,500 ms pulsed 226 Hz probe tone was presented, and middle-ear pressure and compliance readings were recorded. Participants were excluded from the study if they were classified with results different than type A in both ears, based on Jerger’s classification (Jerger, 1970): middle ear compliance < 0.2 cc or middle ear pressure < –150 daPa (decaPascals).

Air-conduction pure-tone thresholds were obtained bilaterally with an Interacoustics AC629 clinical audiometer (Middelfart, Denmark) and Sennheiser HDA 300 headphones. The Hughson-Westlake procedure described by Carhart and Jerger (1959) was used. Hearing thresholds at 0.5, 1, 2, 3, 4, 6, 8, 9, 10, 11.2, 12, and 14 kHz were obtained. Included participants presented with hearing thresholds from 0.5 to 8 kHz, equal to or better than 20 dB HL in at least one ear.

#### Distortion product otoacoustic emissions

Distortion product otoacoustic emissions for both ears were obtained, measured, and analyzed using an Interacoustics Titan equipment with DPOAE440 module (Middlelfart, Denmark), connected to a Lenovo laptop computer (Beijing, China). The primary frequencies selected for the evaluation were the geometric means of *f*_1_ and *f*_2_ at 2, 3, 4, 5, 6, 7, 8, 9, and 10 kHz, using primary levels (L1/L2) of 65/55 dB SPL and a primary ratio (*f*_2_/*f*_1_) of 1.22. The levels of the 2*f*_1_-*f*_2_ DPOAEs and the corresponding noise floor were registered as a function of *f*_2_. Values for DPOAEs were obtained by subtracting the noise floor from the DPOAE amplitudes. Selected participants should have exhibited presence of DPOAEs (+3 dB SNR) for each of the aforementioned frequencies in at least one ear.

#### Auditory brainstem response

The ABR was recorded using an Intelligent Hearing System (IHS, Smart EP model, Miami, FL, United States) connected to a Lenovo laptop (Beijing, China). Surface electrodes were placed at the vertex (Cz, non-inverting electrode) and the forehead (Fpz, ground), in accordance with the International 10–20 system of EEG recordings. In addition, an extra-tympanic electrode (Lilly TM-Wick, IHS, Miami, FL, United States) was placed in the ipsilateral external auditory canal, sitting at the tympanic membrane (inverting electrode). This placement was chosen to improve the visualization of wave I and reduce intra-subject variability (Lefler et al., 2021). The amplifier bandpass was set between 0.3 and 3 kHz. Two trials, each averaging 2,000 responses, were obtained using rarefaction click stimulus at 90, 80, 70, 60, and 50 dBnHL presented monaurally to the better ear (according to pure-tone audiometry and DPOAEs) at a rate of 11.1 stimuli/second, with ER3A insert earphones. Trials were compared to check the reproducibility of the responses. Electrode impedance was less than 5 kOhms. Responses with an amplitude above 30 μV were automatically rejected. In addition, electrical activity/noise that was common to both electrodes (i.e., inverting and non-inverting) was canceled out by common mode rejection. At each stimulus level, when waves I and V were below the residual noise, the waveform was excluded from the analysis. The recordings were visually inspected by a group-blind experienced audiologist to identify waves I, III, and V. The peak-to-trough amplitudes for waves I and V were obtained for analysis purposes. In addition, the slope of the wave I amplitude growth as a function of stimulus intensity (μV/dB) was calculated (AMP-I_Slope_). The AMP-I_Slope_ was computed by fitting a straight line across the conditions in which the waveforms were identifiable. All conditions in which the ABR wave I was clear were required for the linear fits. When this was not the case, the participant was excluded from the analyses. Finally, the wave I/V amplitude ratio for 90 nHL stimulus was obtained in each participant.

In addition, ABRs for rarefaction click stimulus at 80 dBnHL with ipsilateral white noise at 45, 55, 65, 75, and 85 dB SPL were obtained (using a similar method as the one described by Mehraei et al., 2016). Surface electrodes were placed at the scalp, at the vertex (Cz), the ipsilateral mastoid (A1/A2), and the forehead (Fpz, ground). Latencies for wave V with ipsilateral masking noise at each of the aforementioned intensities were obtained. The latency shift (ms/dB) was calculated by fitting a straight line across the conditions in which the waveforms were identifiable at each level of the ipsilateral masking noise (LAT-V_Slope_). All conditions in which the ABR wave V was clear were required for the linear fits. When this was not the case, the participant was excluded from the analyses.

#### Mandarin Bamford-Kowal-Bench sentence test (Mandarin BKB)

Speech recognition in noise was evaluated with the Mandarin BKB (Xi et al., 2012) in the better ear (according to pure-tone audiometry and DPOAEs). Initially, one list of 10 sentences was used as a practice round. Then, two lists of 10 sentences were presented monaurally through HDA 300 headphones (Sennheiser, Germany) at 70 dB HL fixed speech level in a background of four-talker babble noise (three females and one male). For each list, SNRs varied from +21 dB to –6 dB, beginning with the most favorable SNR (+21 dB) and progressing in 3 dB steps to more difficult SNRs (+21, +18, +15, +12, +9, +6, +3, 0, –3, and –6 dB). The first sentence had 4 key words, and the remaining nine sentences each had three key words. Participants were instructed to repeat back each sentence. The number of correctly repeated key words for each list was summed, and afterward subtracted from 23.5 to obtain the SNR-50%. This represents the SNR at which a listener correctly identifies 50% of the key words. Then, an average between SNR-50% for both lists was calculated (Etymotic Research Inc, Elk Grove Village, IL, United States). A lower SNR-50% score indicates better SPiN performance.

### Statistical analysis

Statistical analyses were performed with SPSS V27 (IBM Corp, 2020). First, Student *t*-tests were used to compare the noise-exposed and control groups’ age, and to compare noise levels (CNE/L_Aeq.8h_) between participants who reported to use HPDs and those who did not.

Second, differences in individual hearing thresholds and DPOAE amplitudes were analyzed using repeated measures ANOVAs, with individual frequency as an intra-subject factor and group as a between-subject factor. *Post hoc* Student *t*-tests with Bonferroni corrections were used to describe possible interactions and main effects. Since group differences were observed for pure-tone thresholds at 0.5, 1, and 4 kHz and for extended high frequencies at 11.2, 12, and 14 kHz, two averages were calculated for the hearing thresholds in the better ear: one average for hearing thresholds at 0.5, 1, 2, and 4 kHz (PTA_4_) and another for hearing thresholds from 9 to 14 kHz (PTA_EHF_). Also, a DPOAE_mean_ was calculated by averaging the amplitude in dB SNR of DPOAEs in the better ear across all frequencies (2–10 kHz).

Third, ABR measures (waves I and V amplitudes and latencies, ABR I/V amplitude ratio, AMP-I_Slope_, and LAT-V_Slope_) and BKB test results were compared between groups using Student *t*-tests. An ANCOVA test was also performed for all ABR and BKB measures, controlling for hearing thresholds (PTA_4_ and PTA_EHF_). This aimed to better control for differences in the audiogram between the noise-exposed and control groups.

Pearson correlations between CNE/CNE-HPD, L_Aeq.8h_/L_Aeq.8h_-HPD, age, PTA_4_, PTA_EHF_, DPOAE_mean_, ABR results, and SPiN were computed with the data obtained from the sample of workers exposed to noise. Finally, bivariate and multivariate regression models were constructed to independently investigate possible associations between SPiN (i.e., the dependent variable) and the independent factors of age, PTA_4_, PTA_EHF_, DPOAE_mean_, and the ABR results. For the multivariate models, a backward elimination technique was used to select the remaining significant variables in the adjusted analysis, using a selection criterion of α < 0.05.

## Results

### Age, noise exposure and use of hearing protection devices

The group mean age was 28.4 ± 5.3 years for noise-exposed participants and 21.1 ± 3.7 years for control group participants. Control group participants were significantly younger than noise-exposed participants [*t*(78) = 7.24, *p* < 0.001]. In the noise-exposed group (*n* = 40), the mean occupational noise exposure level for L_Aeq.8h_ was 89.8 ± 5.4 dBA and the group mean of CNE was 96.3 ± 5.6 units of noise exposure (dBA.year), while the L_Aeq.8h_-HPD and the CNE-HPD were 76.7 ± 4.8 dBA and 82.5 ± 5.5 dBA.year respectively. Duration of exposure to noise in the workplace ranged from 2 to 18 years (mean ± SD: 6.3 ± 4.6 years).

Regarding HPDs, 75% of noise-exposed participants (*n* = 30) reported to use them in their workplace. Out of these participants, 90% (*n* = 27) reported to use them “often,” while 10% (*n* = 3) reported to use them “sometimes.” A significantly higher L_Aeq.8h_ [*t*(38) = –2.86, *p* = 0.007] was obtained in participants who reported to use HPDs (91.1 ± 5.4 dBA) compared to those who did not report to use HPDs (85.9 ± 4.5 dBA). A similar result was obtained for CNE [*t*(38) = –2.24, *p* = 0.031], with a higher CNE in participants who reported to use HPDs (97.4 ± 5.2 dBA.year) compared to those who did not (93.0 ± 5.8 dBA.year).

### Hearing thresholds and distortion product otoacoustic emissions

**FIGURE 1:**
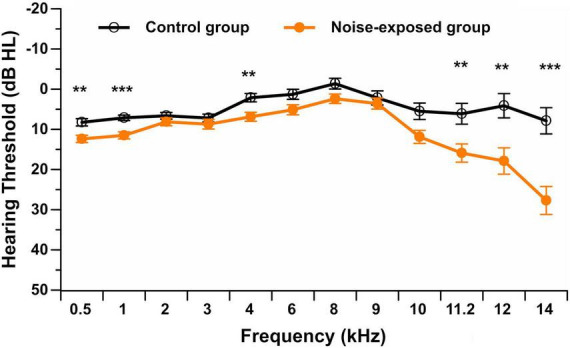
Pure-tone audiometric thresholds (in dB HL) in the better ear from 0.5 to 14 kHz in noise-exposed and control group participants. Error bars represent the standard error. **p < 0.01; ***p < 0.001.

All participants presented with hearing thresholds from 0.5 to 8 kHz, equal to or better than 20 dB HL in the better ear. Note that this was part of the inclusion criteria. Participants also presented with normal or near-normal hearing thresholds in the contralateral ear (equal to or better than 20 dB HL). Figure 1 displays the hearing thresholds in the better ear (0.5–14 kHz) for each group of participants at all tested frequencies.

For the standard pure-tone audiometry (0.5–8 kHz), the repeated measures ANOVA showed no interaction between group and stimulus frequency [*F*(6,450) = 1.35, *p* = 0.232]. A significant main effect of group [*F*(1,75) = 10.06, *p* = 0.002] was observed. *Post hoc t*-tests showed that control group participants presented with a significantly lower (i.e., better) hearing threshold than noise-exposed participants at 0.5 kHz (*p* = 0.002, mean difference of 4.1 dB HL), 1 kHz (*p* < 0.001, mean difference of 4.4 dB HL) and 4 kHz (*p* = 0.003, mean difference of 4.8 dB HL) after controlling for multiple comparisons (Bonferroni correction; 0.05/7 = 0.007). Although statistically reliable, these threshold differences were small and were not clinically significant.

For extended high-frequency pure-tone audiometry (9–14 kHz), the repeated measures ANOVA showed a significant interaction between group and stimulus frequency [*F*(4,304) = 9.91, *p* < 0.001]. *Post hoc t*-tests with a Bonferroni correction showed that participants in the control group did not exhibit significant differences in hearing thresholds among extended high frequencies. However, noise-exposed participants presented with worse hearing thresholds at 14 kHz and better hearing thresholds at 9 kHz compared to all other extended high frequencies (*p* < 0.001). Additionally, control group participants presented with a significantly lower (i.e., better) hearing threshold than noise-exposed participants at 11.2 kHz (*p* = 0.007, mean difference of 9.8 dB HL), 12 kHz (*p* = 0.003, mean difference of 13.8 dB HL), and 14 kHz (*p* < 0.001, mean difference of 19.8 dB HL) after controlling for multiple comparisons (Bonferroni correction; 0.05/5 = 0.01).

**FIGURE 2:**
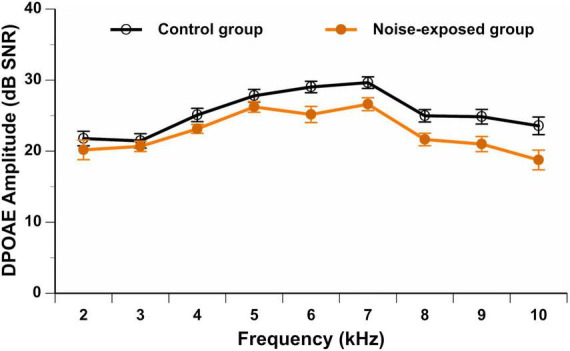
DPOAE amplitudes (in dB SNR) in the better ear from 2 to 10 kHz in noise-exposed and control group participants. Error bars represent the standard error. No significant differences between groups are observed after Bonferroni correction for multiple comparisons.

As previously mentioned, all participants should have presented with DPOAE amplitudes equal to or better than 3 dB SNR at each tested frequency (*f*2: 2–10 kHz) in the better ear. None of the participants presented with an absence of DPOAEs in the contralateral ear (defined as an amplitude smaller than 3 dB SNR). Figure 2 displays the DPOAE amplitudes in the better ear for both groups. The repeated measures ANOVA showed no significant interaction between group and stimulus frequency [*F*(9,666) = 1.68, *p* = 0.089]. A significant main effect of group was observed [*F*(1,74) = 6.36, *p* = 0.014], with control group participants presenting with higher (i.e., better) DPOAE amplitudes than noise-exposed participants. *Post hoc t*-tests showed a significant difference in DPOAE amplitudes between groups at 6 kHz (*p* = 0.007, mean difference of 3.4 dB SNR), 7 kHz (*p* = 0.017, mean difference of 3.0 dB SNR), 8 kHz (*p* = 0.010, mean difference of 3.3 dB SNR), 9 kHz (*p* = 0.013, mean difference of 3.9 dB SNR) and 10 kHz (*p* = 0.013, mean difference of 4.8 dB SNR). However, these differences were no longer significant after controlling for multiple comparisons (Bonferroni correction; 0.05/10 = 0.005).

### Auditory brainstem response

**FIGURE 3:**
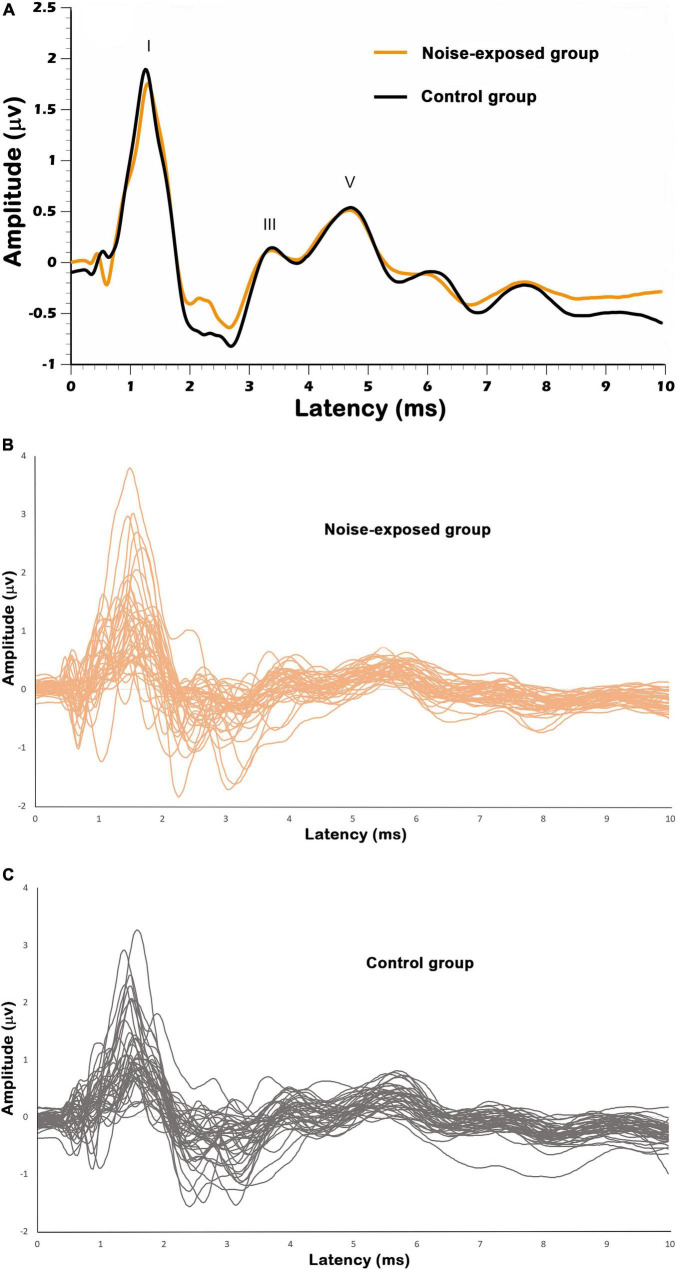
**(A)** Grand mean ABR triggered by click stimulus at 90 dBnHL for the noise-exposed and control group. The individual ABR waveforms are also illustrated in **(B,C)** for noise-exposed and control groups. Surface electrodes were placed at the vertex (Cz, non-inverting electrode) and the forehead (Fpz, ground), while an extra-tympanic electrode (inverting electrode) was placed sitting at the tympanic membrane. I, III, and V denote wave I, wave III, and wave V.

Figure 3A displays the grand mean ABR waveform for each group of participants, which was obtained using click stimuli at 90 dBnHL. In Figures 3B,C, individual ABR waveforms for click stimulus at 90 dBnHL are displayed for noise-exposed and control participants respectively.

Peak-to-trough amplitudes (μV) and latencies (ms) for ABR wave I and wave V at each stimulus presentation level (i.e., 90, 80, 70, 60, and 50 dBnHL) were obtained for each participant (see Table 1 for a summary). Even at low stimulus levels, waves I and V were identifiable in most waveforms and the response was above the residual noise (e.g., at 50 dBnHL, *n* = 67 for wave I and *n* = 76 for wave V; at 60 dBnHL, *n* = 68 for wave I and *n* = 74 for wave V; at 70 dBnHL, *n* = 78 for waves I and V). Mean wave I amplitudes ranged from 0.27 μV at 50 dB nHL to 1.88 μV at 90 dB nHL.

**TABLE 1:** Mean, standard deviation, and group comparisons for ABR wave I and wave V variables (amplitude and latency).

	Noise-exposed group	Control group	
ABR measures	Mean ± SD (*n*)	Mean ± SD (*n*)	*P*-value
**Amplitude (μV)**			
**Wave I**			
90 dBnHL	1.72 ± 1.00 (*n* = 40)	1.88 ± 1.12 (*n* = 40)	0.513
80 dBnHL	1.45 ± 0.91 (*n* = 36)	1.38 ± 0.82 (*n* = 40)	0.700
70 dBnHL	0.76 ± 0.53 (*n* = 38)	0.83 ± 0.51 (*n* = 40)	0.568
60 dBnHL	0.35 ± 0.34 (*n* = 34)	0.33 ± 0.23 (*n* = 34)	0.786
50 dBnHL	0.27 ± 0.35 (*n* = 34)	0.35 ± 0.28 (*n* = 33)	0.335
**Wave V**			
90 dBnHL	0.51 ± 0.19 (*n* = 40)	0.54 ± 0.25 (*n* = 40)	0.521
80 dBnHL	0.40 ± 0.19 (*n* = 36)	0.42 ± 0.16 (*n* = 40)	0.484
70 dBnHL	0.32 ± 0.13 (*n* = 39)	0.34 ± 0.18 (*n* = 39)	0.650
60 dBnHL	0.24 ± 0.09 (*n* = 36)	0.26 ± 0.14 (*n* = 38)	0.593
50 dBnHL	0.21 ± 0.10 (*n* = 40)	0.22 ± 0.09 (*n* = 36)	0.487
**Latency (ms)**			
**Wave I**			
90 dBnHL	1.56 ± 0.17 (*n* = 40)	1.54 ± 0.16 (*n* = 40)	0.615
80 dBnHL	1.68 ± 0.18 (*n* = 36)	1.64 ± 0.16 (*n* = 40)	0.289
70 dBnHL	1.88 ± 0.29 (*n* = 38)	1.83 ± 0.22 (*n* = 40)	0.383
60 dBnHL	2.20 ± 0.34 (*n* = 34)	2.17 ± 0.33 (*n* = 34)	0.687
50 dBnHL	2.74 ± 0.38 (*n* = 34)	2.61 ± 0.30 (*n* = 33)	0.128
**Wave V**			
90 dBnHL	5.65 ± 0.24 (*n* = 40)	5.59 ± 0.23 (*n* = 40)	0.263
80 dBnHL	5.75 ± 0.22 (*n* = 36)	5.71 ± 0.22 (*n* = 40)	0.393
70 dBnHL	5.91 ± 0.26 (*n* = 39)	5.85 ± 0.23 (*n* = 39)	0.299
60 dBnHL	6.20 ± 0.36 (*n* = 36)	6.10 ± 0.27 (*n* = 38)	0.188
50 dBnHL	6.56 ± 0.39 (*n* = 40)	6.52 ± 0.27 (*n* = 36)	0.571

**FIGURE 4:**
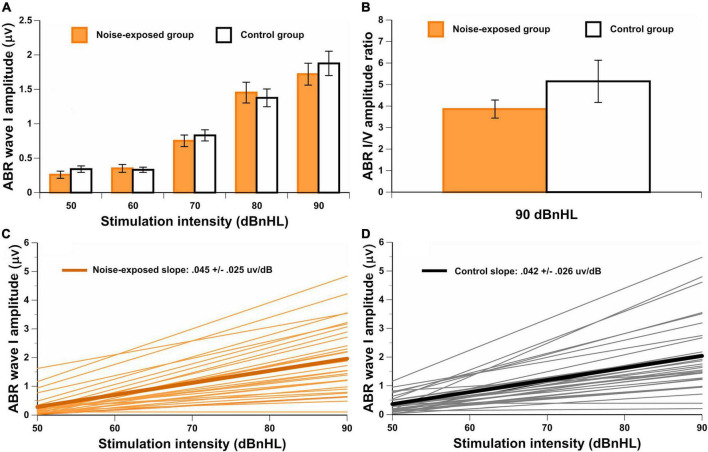
**(A)** ABR wave I amplitude for both groups at each stimuli level. In **(B)**, the wave I/V amplitude ratio for both groups at 90 dBnHL. The group mean and individual results for the ABR AMP-I_Slope_ are also illustrated in **(C,D)** for noise-exposed and control groups. There are no significant differences between groups.

No significant differences between groups were observed for the amplitudes of wave I at 50 dBnHL [*t*(65) = –0.97, *p* = 0.335], 60 dBnHL [*t*(66) = 0.27, *p* = 0.786], 70 dBnHL [*t*(76) = –0.57, *p* = 0.568], 80 dBnHL [*t*(74) = 0.39, *p* = 0.700] and 90 dBnHL [*t*(78) = –0.66, *p* = 0.513]. Similarly, no differences between groups were observed for the amplitudes of wave V at 50 dBnHL [*t*(74) = –0.70, *p* = 0.487], 60 dBnHL [*t*(72) = –0.54, *p* = 0.593], 70 dBnHL [*t*(76) = –0.46, *p* = 0.650], 80 dBnHL [*t*(74) = –0.70, *p* = 0.484] and 90 dBnHL [*t*(74) = –0.96, *p* = 0.342] (see Table 1). Wave I amplitudes for both groups at each stimulus level are also shown in Figure 4A. The ABR I/V amplitude ratio at 90 dBnHL was calculated for each participant to better control for individual variability (Figure 4B). No significant difference between noise-exposed participants and control participants was observed [*t*(78) = –1.21, *p* = 0.230]. Regarding the ABR wave I and V latencies, no significant differences were observed between groups at any stimulus levels (see Table 1).

Additionally, the ABR AMP-I_Slope_ was computed. For some participants, the ABR AMP-I_Slope_ could not be obtained because the waveform was not identified in at least one stimulus level (*n* = 9 for the noise-exposed group and *n* = 9 for the control group). No significant difference between groups was observed for the ABR AMP-I_Slope_ [*t*(60) = –0.02, *p* = 0.984] (Figures 4C,D).

**FIGURE 5:**
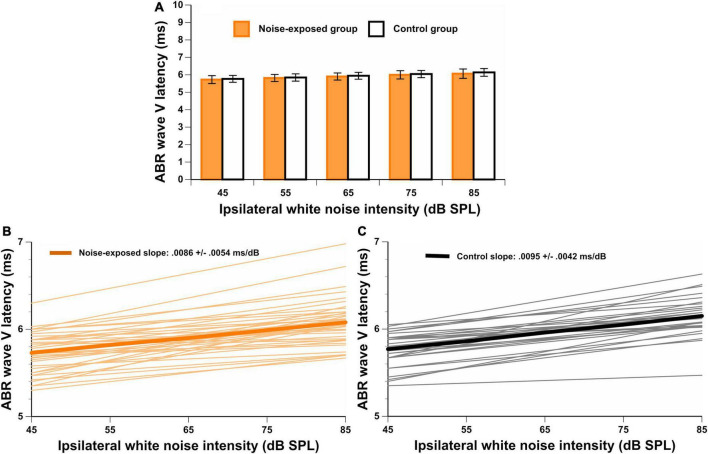
**(A)** Wave V latencies for a stimulation level of 80 dBnHL with different ipsilateral white noise intensities (45, 55, 65, 75, and 85 dB SPL). In **(B,C)**, the ABR LAT-V_Slope_ is illustrated for both groups. There are no significant differences between groups.

Finally, the ABR LAT-V_Slope_ was obtained for each participant. Figure 5A displays the ABR wave V latency at each intensity level of the ipsilateral white noise. In Figures 5B,C, the ABR wave V latency shift as a function of ipsilateral white noise intensity (ABR LAT-V_Slope_) is displayed for noise-exposed and control participants respectively. The response was above the residual noise for all recordings, and the wave V with ipsilateral white noise was identifiable in most waveforms. For some participants, the ABR LAT-V_Slope_ could not be calculated because the waveform was not identified for at least one intensity level of the white noise (*n* = 2 for the noise-exposed group and *n* = 10 for the control group). No significant difference between groups for the ABR LAT-V_Slope_ was observed [*t*(66) = –0.66, *p* = 0.514]. In addition to these analyses, we performed another analysis on ABR outcomes between groups controlling for PTA_4_ and PTA_EHF_ (see Supplementary Table 1). No significant differences between groups were observed for any ABR outcomes.

### Speech perception in noise

**FIGURE 6:**
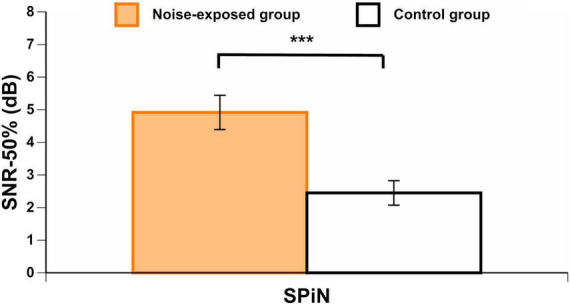
BKB scores in each group. Higher scores indicate a higher signal-to-noise ratio loss (worse SPiN performance). Error bars represent the standard error. ***p < 0.001.

Noise-exposed participants presented with significantly poorer BKB results (i.e., higher SNR-50%) than control group participants [*t*(73) = 3.87, *p* < 0.001] (Figure 6), even when controlling for PTA_4_ and PTA_EHF_ by using an ANCOVA [*F*(1,71) = 6.55, *p* = 0.013].

### Correlations between noise exposure and auditory outcomes

A Pearson correlation matrix between CNE/CNE-HPD, L_Aeq.8h_/L_Aeq.8h_-HPD, hearing thresholds (PTA_4_ and PTA_EHF_), DPOAE_mean_, ABR results, and BKB scores was obtained in noise-exposed participants (*n* = 40) (Table 2). First, no significant correlations were observed between CNE/L_Aeq.8h_ (uncorrected and corrected for HPD use) variables and any of the auditory outcomes (PTA_4_, PTA_EHF_, DPOAE_mean_, ABR and BKB results). Second, DPOAE_mean_ was significantly correlated with age, PTA_EHF_, and ABR I/V amplitude ratio. Third, the amplitude of wave I at 90 dBnHL was significantly correlated with the ABR I/V amplitude ratio and the AMP-I_Slope_. Finally, BKB results were not correlated with the ABR measures.

**TABLE 2:** Correlation coefficients (Pearson) between L_Aeq.8h/_L_Aeq.8h_-HPD, CNE/CNE-HPD, age, hearing thresholds, DPOAEs, BKB results, and ABR measures for the noise-exposed group.

	Age	PTA_4_	PTA_EHF_	DPOAE_mean_	BKB	Amp I	I/V	AMP-I_Slope_	LAT-V_Slope_
L_Aeq.8h_	–0.178	0.060	–0.031	0.059	–0.159	0.186	0.120	0.342	0.095
L_Aeq.8h_-HPD	–0.273	0.140	0.004	0.041	–0.157	0.140	0.083	0.337	0.086
CNE	0.181	0.137	–0.073	0.034	–0.137	0.285	0.182	0.342	0.177
CNE-HPD	0.106	0.254	0.012	–0.008	–0.152	0.136	0.059	0.326	0.146
Age	⋅	–0.072	0.200	–0.413**	–0.057	–0.019	0.063	–0.110	–0.024
PTA_4_	⋅	⋅	0.044	0.149	0.266	–0.280	–0.131	–0.216	0.114
PTA_EHF_	⋅	⋅	⋅	–0.425**	0.008	–0.095	–0.057	–0.071	–0.005
DPOAE_mean_	⋅	⋅	⋅	⋅	–0.122	–0.236	–0.397*	–0.138	–0.082
BKB	⋅	⋅	⋅	⋅	⋅	–0.265	–0.021	–0.309	–0.168
Amp I	⋅	⋅	⋅	⋅	⋅	⋅	0.777***	0.960***	0.272
I/V	⋅	⋅	⋅	⋅	⋅	⋅	⋅	0.685***	0.161
AMP-I_Slope_	⋅	⋅	⋅	⋅	⋅	⋅	⋅	⋅	0.327
LAT-V_Slope_	⋅	⋅	⋅	⋅	⋅	⋅	⋅	⋅	⋅

L_Aeq.8h_, equivalent continuous sound level for an 8 h work shift; L_Aeq.8h_-HPD, equivalent continuous sound level for an 8 h work shift corrected for HPD use; CNE, cumulative noise exposure; CNE-HPD, cumulative noise exposure corrected for HPD use; Age, age of participant in years; PTA_4_, pure-tone threshold average of the better ear at 0.5, 1, 2, and 4 kHz; PTA_EHF_, pure-tone threshold average of the better ear from 9 to 14 kHz; DPOAE_mean_, DPOAEs amplitudes of the better ear from 2 to 10 kHz; BKB, Mandarin Bamford-Kowal-Bench sentence test scores; Amp I, ABR wave I amplitude at 90 dBnHL (μV); I/V, amplitude ratio between ABR wave I and wave V at 90 dBnHL; AMP-I_Slope_, slope of the ABR wave I amplitude growth as a function of stimulus intensity; LAT-V_Slope_, shift of the ABR wave V latency with ipsilateral white noise. *p < 0.05; **p < 0.01; ***p < 0.001.

### Regression models

The previous analyses showed significant differences between noise-exposed workers and control group participants for BKB scores. Therefore, bivariate linear regression analyses were carried out to further examine associations between BKB scores and group category (noise exposure) along with other factors that may be associated with SPiN including both age and auditory outcomes (PTA_4_, PTA_EHF_, DPOAE_mean_, and ABR AMP-I_Slope_). Then, multivariate regression analyses were performed to model the association between BKB scores and the factors tested in the bivariate regression models (Table 3). Age, group category (noise exposure) and PTA_4_ were significantly associated with BKB scores in the bivariate models. The final multivariate regression model indicated that group category (noise exposure) and PTA_4_ significantly predicted 23.3% of the variability in the BKB scores.

**TABLE 3:** Bivariate and multivariate linear regression analyses for BKB scores.

	Bivariate model			Initial multivariate model		Final multivariate model	
Characteristic	Beta	*P*-value	*R* ^2^	Beta	*P*-value	Beta	*p*-value
Age	0.295	0.010	0.087	–0.077	0.616		
Occupational noise exposure: Exposed	0.413	<0.001	0.171	0.318	0.034	0.297	0.009
Unexposed	Ref						
DPOAE_mean_	–0.270	0.019	0.073	–0.370	0.019		
PTA_4_	0.422	<0.001	0.178	0.312	0.015	0.311	0.006
PTA_EHF_	0.226	0.051	0.051	–0.206	0.177		
ABR AMP-I_Slope_	–0.212	0.104	0.045	–0.198	0.090		
				Adjusted *R*^2^ = 0.272		Adjusted *R*^2^ = 0.233	

Age, age of participant in years; PTA_4_, pure-tone threshold average of the better ear at 0.5, 1, 2, and 4 kHz; PTA_EHF_, pure-tone threshold average of the better ear from 9 to 14 kHz; DPOAE_mean_, DPOAEs amplitudes of the better ear from 2 to 10 kHz; ABR AMP-I_Slope_, slope of the ABR wave I amplitude growth as a function of stimulus intensity.

## Discussion

### Auditory brainstem response outcomes

In this study, we used four ABR outcomes that may be affected by cochlear synaptopathy. Occupationally noise-exposed and control participants did not significantly differ for any of these outcomes (i.e., wave I amplitude at 90 dBnHL, wave I/V amplitude ratio at 90 dBnHL, the slope of the wave I amplitude growth as a function of stimulus intensity, and the slope of wave V latency shift as a function of ipsilateral white noise intensity). To our knowledge, this was the first study investigating all four ABR outcomes in normal hearing young adults with occupational noise exposure. The results indicate that cochlear synaptopathy was not observed in this sample of participants, or that these ABR outcomes were not sensitive enough to detect this condition in humans with the characteristics of our sample.

Previous studies in humans have extensively used the ABR in an attempt to detect cochlear synaptopathy in humans. Like this study, other studies investigating non-occupational populations exposed to noise have not found an effect of noise exposure on ABR wave I amplitude (Fulbright et al., 2017; Prendergast et al., 2017a; Ridley et al., 2018; Couth et al., 2020). Additionally, in a study with 20 normal hearing persons with occupational noise exposure, Pushpalatha and Konadath (2016) did not find an effect of noise exposure on ABR wave I amplitude. However, a reduction of ABR wave I amplitude associated with noise exposure has been found by some researchers in non-occupational samples of persons exposed to noise (Stamper and Johnson, 2015; Valderrama et al., 2018; Wang et al., 2021). In addition, a reduction in wave I amplitude was observed in a population of veterans exposed to firearms (Bramhall et al., 2017) and in a population of musicians (Kikidis et al., 2020). A number of factors, such as participants’ inclusion criteria, noise exposure metrics, and participants’ profiles, may explain the differences in study results. In two of these studies (Valderrama et al., 2018; Kikidis et al., 2020), researchers did not control for possible hair cell deficits (measured by hearing thresholds and DPOAEs), which could likely explain the reduced ABR wave I amplitude in the noise-exposed group.

The differences in results among the previous studies may also be explained by intersubject variability of ABR wave I amplitude due to electrode placement and head size (Bramhall, 2021). Therefore, it has been suggested that using the ABR wave I/V amplitude ratio can diminish that variability by canceling out the subject-specific factors that impact all peaks. However, when the measure was used in this research, no significant differences between groups were observed. Like this study, previous research has not found an association between noise exposure and the ABR wave I/V amplitude ratio (Guest et al., 2017). However, other authors have reported a reduced ABR wave I/V amplitude ratio associated with non-occupational noise exposure (Grose et al., 2017) or tinnitus (Schaette and McAlpine, 2011). This reduced ratio has been explained by a smaller wave I amplitude with no changes in wave V amplitude. It is important to note that some of the studies that have found an effect of noise exposure on ABR wave I and/or wave I/V amplitude ratio have included female participants. It has been reported that gender has an effect on ABR outcomes (for a review, see Bramhall, 2021), and that may have affected their results. In this study, we selected only male workers, with the aim of controlling for gender differences in ABR. Finally, the stimulation rate may be another explanation for the divergent results among studies. Kikidis et al. (2020) found a reduced ABR wave I amplitude and I/V amplitude ratio in musicians compared to non-musicians, and such differences were more marked at higher stimulation rates. The authors concluded that a higher stimulation rate would better allow the detection of cochlear synaptopathy. However, the reasoning of Kikidis and colleagues’ rests on the assumption that low-SR fibers will be “stressed” by high presentation rates. It could be argued more cogently that high presentation rates will reduce the contribution of low-SR fibers to the response, leading to ABRs that are dominated by high-SR fibers and hence less sensitive to cochlear synaptopathy.

In this study, we also calculated the slope of ABR wave I amplitude growth as a function of stimulus intensity (AMP-I_Slope_). We hypothesized that in the presence of cochlear synaptopathy, noise-exposed workers would present with a reduced AMP-I_Slope_ as compared to unexposed participants. This was because at low stimulation intensity, the activity of the auditory system mainly comes from medium- and high-SR fibers, which are less susceptible to noise exposure (Bourien et al., 2014; Marmel et al., 2015). As the stimulus intensity increases, the auditory system also increases the recruitment of low-SR auditory fibers, which are affected by cochlear synaptopathy (Furman et al., 2013). Thus, individuals with cochlear synaptopathy should exhibit a reduced AMP-I_Slope_ as compared to individuals who do not exhibit cochlear synaptopathy. The results of this study did not support our hypothesis, as no differences between the noise-exposed and control participants were found. Previously, Bramhall et al. (2020) found a steeper ABR wave I amplitude growth function in veterans with decreased sound tolerance. However, this finding was observed in a different population (i.e., veterans with exposure to impulse noise from firearms and with reported decreased sound tolerance) than the one investigated in this study.

Finally, we obtained the latency of ABR wave V in the presence of ipsilateral white noise at different intensities. The aim of this technique was to obtain the slope of the amount of shift of ABR wave V latency as a function of the intensity of the masker (LAT-V_Slope_). We hypothesized that in the presence of cochlear synaptopathy, noise-exposed workers would present with a reduced LAT-V_Slope_ compared to unexposed participants. This hypothesis was supported by the results of Mehraei et al. (2016). They found that mice with histologically confirmed cochlear synaptopathy showed a smaller latency shift of wave IV (equivalent to wave V in humans) in the presence of masking noise than control mice. In addition, in their human cohort, they found that participants with reduced wave V latency shift also displayed worse performances on a sound localization in noise task. However, since Mehraei et al. (2016) did not quantify participants’ noise exposure, it is still unclear if this ABR outcome might be affected by cochlear synaptopathy in humans. In our study, we did not find significant differences between groups for this ABR outcome. To our knowledge, no other studies have used this technique to detect cochlear synaptopathy in humans exposed to noise.

In addition to group comparisons, we performed a correlation analysis with noise-exposed workers between their noise exposure levels and auditory outcomes (e.g., ABR and SPiN). Noise exposure levels (i.e., L_Aeq.8h_ and CNE, corrected and uncorrected for HPD use) were not significantly correlated with these outcomes. Also, BKB scores, which showed significant differences between groups (see below), were not significantly correlated with the ABR outcomes used in this study. In addition, note that noise-exposed workers were significantly older (by around 7 years) than control participants. They also presented with significantly worse hearing thresholds at some frequencies than control participants, although these were within normal ranges. Both variables are likely to reduce ABR wave I amplitude, and yet no significant differences between groups were observed. In light of these results, we believe that cochlear synaptopathy could not be observed in this sample of workers.

### Speech perception in noise

Significantly worse SPiN scores (BKB) were found for noise-exposed participants than for controls. These results were in agreement with those of some previous studies conducted on university students (Liberman et al., 2016) and construction workers (Vijayasarathy et al., 2021). For example, Liberman et al. (2016) found significantly worse results for SPiN in individuals considered at high risk to develop cochlear synaptopathy (based on their noise exposure history) than in individuals considered at low risk. However, these results were only obtained for the most challenging listening conditions (with reverberation and time-compressed speech). Furthermore, the SPiN material was presented at moderate (around 40 dB SPL) intensity, where it is unclear how much recruitment of low-SR fibers there would be. Several other studies have not found an effect of noise exposure on SPiN outcomes (e.g., Fulbright et al., 2017; Grinn et al., 2017; Grose et al., 2017; Prendergast et al., 2017b; Yeend et al., 2017; Guest et al., 2018; Smith et al., 2019).

In addition to the significant difference between groups for BKB scores, a multivariate regression model showed that 23.3% of the variance in BKB scores was explained by group category (noise-exposed vs. control) and PTA_4_. Age, DPOAE_mean_, PTA_EHF_, and ABR AMP-I_Slope_ did not explain the worse SPiN scores in the noise-exposed group, since these factors were not associated with BKB scores in the regression model. Audibility has been suggested to be associated with performance on SPiN tests, although it does not fully account for the variance in SPiN scores (e.g., Anderson et al., 2011). In this study, we hypothesized that workers exposed to noise would exhibit signs of cochlear synaptopathy. However, we discard the hypothesis that cochlear synaptopathy explains the effects of group category on BKB scores. This is because, as discussed previously, no signs of cochlear synaptopathy were observed in the sample of workers exposed to noise by the use of four ABR outcomes. In addition, noise exposure levels (L_Aeq.8h_ and CNE, corrected and uncorrected for HPD use) were not significantly associated with BKB scores. Therefore, we suggest that variables associated with group category other than noise exposure may explain these results. Factors that go beyond sensory processing may have been implicated. For example, factory workers are likely to present with poorer performance for working memory, attention, and language capacities than university students (control group participants). This is because in general factory workers in China have a lower educational level (Chen and Guan, 2016). It has been previously reported that both cognitive resources and language competence can influence SPiN performance (Schneider et al., 2002; Pienkowski, 2017; DiNino et al., 2022). These aspects were not explored in the present study, and thus, we cannot conclude that sensory processing was the main underlying factor that explained our results. Future studies should control for cognitive abilities when interpreting SPiN performance in individuals with occupational noise exposure. In summary, we conclude that differences between groups for BKB scores were not likely associated with cochlear synaptopathy or with another auditory deficit associated with noise exposure, but rather that such differences likely rely on non-sensory processing differences between groups.

### Limitations

We identified five main limitations in the present study. First, we collected data from participants’ better ears. For some participants, data were acquired in the left ear, while for others, testing was conducted in the right ear. A recent study suggested that electrophysiological measures (i.e., ABR wave I/V amplitude ratio) are associated with SPiN performance, specifically in the left ear (Megarbane and Fuente, 2020). This could be explained by differences in aspects such as internal redundancy between the right and the left auditory pathways, with the left-ear pathway being less dominant for the processing of speech stimuli than the right-ear pathway (Lazard et al., 2012). It is generally accepted that click ABR latencies are relatively symmetrical between the right and the left ears (Rowe, 1978). However, results regarding ABR amplitudes are less clear, as some researchers have suggested bigger ABR amplitudes for right ear stimulation (Levine et al., 1988). Since we did not control for the tested ear (right versus left) when comparing results between groups, we are not certain whether possible ear asymmetries for the processing of stimuli might have affected our results. Future studies should explore the possible differences between the right and the left ear for the measurement of cochlear synaptopathy in persons occupationally exposed to noise.

Second, the SPiN test (BKB) consisted of the repetition of sentences, which relies on a higher cognitive load than the repetition of words. None of the participants had a cognitive assessment, and young university students likely have better cognitive and language abilities than young factory workers. Note that the BKB speech material was created to be understood by children aged between 4 and 5 years (Xi et al., 2012). This may have decreased the effect of language experience differences between groups in this study. However, differences in cognitive capacities between groups are not controlled by the characteristics of the verbal material in the speech test.

Third, we selected participants with normal hearing thresholds and normal DPOAE amplitudes. This procedure might have caused a selection bias, which could explain the lack of significant differences in some experimental measures and the lack of correlation between these measures and noise exposure variables (L_Aeq.8h_ and CNE). We probably selected people with “tough” ears, who might not have presented evident signs of cochlear synaptopathy. This conclusion is supported by other studies that have found a difference in individual susceptibility to noise, suggesting the idea of “tough” versus “tender” ears (Cody and Robertson, 1983; Maison and Liberman, 2000; Lie et al., 2016). It is possible that individuals with “tough ears” are less susceptible to noise exposure, and will therefore not exhibit poorer hearing outcomes related to cochlear synaptopathy (e.g., ABR). In this study, we might have selected participants who did not present with cochlear synaptopathy, since normal hearing thresholds and DPOAEs were required for participation. However, we believe this to be a reasonable approach to investigating neural damage “beyond the audiogram.”

Fourth, a regular use of HPDs was observed in participants with high noise exposure (>90 dBA). For participants who did not report to use HPDs, noise levels (L_Aeq.8h_/CNE) were significantly lower than for participants who reported to use HPDs. Therefore, it is possible that the regular use of HPDs might have reduced noise exposure and prevented cochlear synaptopathy to develop in our sample of workers. This might explain why no differences in ABR were measured between our groups and why no correlations were observed between L_Aeq.8h_/CNE and other variables used to investigate cochlear synaptopathy (i.e., ABR and SPiN). Although we incorporated HPD use into noise-exposure calculations, HPD reports by participants might not have been accurate enough to estimate the actual noise exposure.

Finally, we tried to control for significant recreational noise exposure, such as exposure to firearms, playing a musical instrument or in a band, frequent attendance to noisy bars and/or nightclubs, along with excessive use of listening devices at elevated volumes. However, since we did not measure recreational noise exposure by dosimetry, we relied on participants’ responses regarding significant noise exposure, which might have been insufficiently sensitive.

## Conclusion

The sample of occupationally noise-exposed participants did not differ from control participants without occupational noise exposure for four ABR outcomes that may detect cochlear synaptopathy (i.e., wave I amplitude at 90 dBnHL, wave I/V amplitude ratio at 90 dBnHL, the slope of the wave I amplitude growth as a function of stimulus intensity, and the slope of wave V latency shift as a function of ipsilateral white noise intensity). Noise-exposed workers exhibited worse SPiN results than control group participants. However, we suggest that factors associated with non-sensory processing are likely to explain such results. The results of the present study suggest that noise exposure was not significantly associated with cochlear synaptopathy in this sample of workers. Further studies are still required to determine whether occupational noise exposure is associated with cochlear synaptopathy prior to observing changes in the audiogram.

## Data availability statement

The raw data supporting the conclusions of this article will be made available by the authors, without undue reservation.

## Ethics statement

The studies involving human participants were reviewed and approved by the Ethics Committee of the Faculty of Medicine of the University of Montreal, the Committee on the Protection of Human Subjects of SUNY Plattsburgh, and the Ethics Committee of Zhejiang Provincial Center for Disease Control and Prevention. The patients/participants provided their written informed consent to participate in this study.

## Author contributions

AP-S wrote the manuscript. WQ and AF designed the study and provided the critical revision of the manuscript. WZ and MZ performed the experiments and collected the data. AP-S, KM-D, and AF analyzed the data. All authors discussed the results and implications and commented on the manuscript at all stages.

## Conflict of interest

The authors declare that the research was conducted in the absence of any commercial or financial relationships that could be construed as a potential conflict of interest.

## Publisher’s note

All claims expressed in this article are solely those of the authors and do not necessarily represent those of their affiliated organizations, or those of the publisher, the editors and the reviewers. Any product that may be evaluated in this article, or claim that may be made by its manufacturer, is not guaranteed or endorsed by the publisher.
