# Supplementary material for: No effect of occupational noise exposure on auditory brainstem response and speech perception in noise
Source: Front Neurosci. 2022 Jul 22;16:915211. doi: 10.3389/fnins.2022.915211 (PMC9354017; doi:10.3389/fnins.2022.915211)
Supplement: Supplementary file 1 [file Table_1.DOCX]

Supplementary Material

Supplementary Table 1. Adjusted group means, standard errors and group comparisons for ABR outcomes, controlled for PTA_4_ and PTA_EHF_ (wave I and wave V amplitude and latency, ABR I/V amplitude ratio at 90 dBnHL, ABR AMP-I_Slope_ and ABR LAT-V_Slope_)

|  | Noise-exposed group | Control group |  |
| --- | --- | --- | --- |
| ABR measures | Mean ± SE | Mean ± SE | p-value |
| Amplitude (µV) |  |  |  |
| Wave I |  |  |  |
| 90 dBnHL | 1.93 ± 0.20 | 1.88 ± 0.20 | p = .887 |
| 80 dBnHL | 1.60 ± 0.17 | 1.42 ± 0.17 | p = .450 |
| 70 dBnHL | 0.92 ± 0.10 | 0.85 ± 0.10 | p = .612 |
| 60 dBnHL | 0.40 ± 0.05 | 0.32 ± 0.05 | p = .311 |
| 50 dBnHL | 0.32 ± 0.06 | 0.33 ± 0.06 | p = .923 |
| Wave V |  |  |  |
| 90 dBnHL | 0.52 ± 0.04 | 0.54 ± 0.04 | p = .637 |
| 80 dBnHL | 0.42 ± 0.03 | 0.40 ± 0.03 | p = .626 |
| 70 dBnHL | 0.34 ± 0.03 | 0.34 ± 0.03 | p = .947 |
| 60 dBnHL | 0.25 ± 0.02 | 0.26 ± 0.02 | p = .849 |
| 50 dBnHL | 0.20 ± 0.02 | 0.22 ± 0.02 | p = .471 |
| Latency (ms) |  |  |  |
| Wave I |  |  |  |
| 90 dBnHL | 1.55 ± 0.03 | 1.52 ± 0.03 | p = .518 |
| 80 dBnHL | 1.68 ± 0.03 | 1.62 ± 0.03 | p = .304 |
| 70 dBnHL | 1.88 ± 0.05 | 1.85 ± 0.05 | p = .751 |
| 60 dBnHL | 2.17 ± 0.06 | 2.21 ± 0.06 | p = .582 |
| 50 dBnHL | 2.68 ± 0.06 | 2.64 ± 0.06 | p = .665 |
| Wave V |  |  |  |
| 90 dBnHL | 5.67 ± 0.04 | 5.58 ± 0.04 | p = .146 |
| 80 dBnHL | 5.75 ± 0.04 | 5.70 ± 0.04 | p = .405 |
| 70 dBnHL | 5.90 ± 0.04 | 5.87 ± 0.04 | p = .658 |
| 60 dBnHL | 6.18 ± 0.06 | 6.13 ± 0.06 | p = .573 |
| 50 dBnHL | 6.56 ± 0.06 | 6.55 ± 0.06 | p = .834 |
| Wave I/V amplitude ratio at 90 dBnHL | 4.45 ± 0.81 | 4.18 ± 0.92 | p = .837 |
| AMP-I_Slope_ (µV/dB) | 0.046 ± 0.005 | 0.040 ± 0.005 | p = .443 |
| LAT-V_Slope_ (ms/dB) | 0.008 ± 0.001 | 0.10 ± 0.001 | p = .398 |
